# First discovery of actinopterygian cutting-edged teeth from the middle Norian (Late Triassic) at the Tulong section, southern Tibet, China

**DOI:** 10.7717/peerj.18728

**Published:** 2024-12-18

**Authors:** Zichen Fang, Long Cheng, Haishui Jiang, Xianlang Wu, Xulong Lai, James G. Ogg

**Affiliations:** 1 Hubei Key Laboratory of Paleontology and Geological Environment Evolution, Wuhan Center of China Geological Survey, Wuhan, Hubei, China; 2 State Key Laboratory of Biogeology and Environmental Geology, School of Earth Sciences, China University of Geosciences, Wuhan, Hubei, China; 3 Purdue University, West Lafayette, United States of America

**Keywords:** Actinopterygii, Birgeria, Himalayasaurus tibetensis, Qulonggongba formation, Tooth morphology

## Abstract

Actinopterygians (ray-finned fishes) successfully passed through the Permian-Triassic Mass Extinction (PTME) and flourished in the Triassic with diverse feeding specializations and occupation of various trophic levels. *Birgeria*, one of the largest actinopterygian fish of the Triassic, was characterized by a strong, blunt rostrum and three rows of sharp cutting-edged teeth, making them the top predators in the Early Mesozoic oceanic ecosystem. These fishes rapidly radiated and diversified globally during the Early and Middle Triassic, but the fossil record is rare for the Neo-Tethys in the Late Triassic. Here, we report new actinopterygian teeth with cutting edges from Norian-age strata in the Tulong section, which was located on the northern margin of the Indian Plate at that time. The tooth features, such as the polished acrodin cap, the ratio of the acrodin cap in length, and the tiny vertical striae at the tooth base, suggest an affinity with *Birgeria*, which is reported in this region for the first time. Furthermore, we infer that the carnivorous *Birgeria*, which co-occurred with the enigmatic ichthyosaur *Himalayasaurus tibetensis*, played the role of predator in this part of the Neo-Tethys marine realm during the Late Triassic. These new findings increase the known diversity of actinopterygians during the Late Triassic and provide further insight into the marine fauna of this epoch.

## Introduction

In the aftermath of the Permian-Triassic Mass Extinction (PTME), the gradual recovery of a complex trophic network led to the during the Early Triassic led to the emergence of large vertebrate predators (Hu et al., 2011; Chen & Benton, 2012; Scheyer et al., 2014). Actinopterygian (ray-finned) fish underwent rapid diversification and different feeding adaptations during the Triassic, and some of these fish, such as *Birgeria* and *Saurichthys*, are considered to have occupied the highest trophic level in some marine ecosystems at that time (Scheyer et al., 2014; Romano et al., 2017; Benton & Wu, 2022; Dai et al., 2023). Because actinopterygians played an important role in the Triassic marine food chain, it is crucial to unravel the evolution of their temporal and spatial distribution for comprehending the development of the modern marine ecosystem (Romano et al., 2014; Smithwick & Stubbs, 2018).

*Birgeria*, a diverse genus encompassing eleven species, is characterized by their massive and elongated body with a strong and blunt rostrum and armed by three rows of sharp cutting-edged teeth, which are indicative of efficient predatory feeding (Lombardo & Tintori, 2005; Romano et al., 2017; Tintori & Lombardo, 2018; Ni et al., 2019). They were widespread and common throughout the Triassic period, with occurrences spanning the Induan through Rhaetian stages (Scheyer et al., 2014; Romano et al., 2017; Ni et al., 2019). During the Early and Middle Triassic, *Birgeria* exhibited a global distribution (Romano et al., 2017; Tintori & Lombardo, 2018). However, in the Late Triassic, the diversity of these fishes was reduced, and there is a gap in the fossil record of marine fish fauna between the early Carnian and the middle to late Norian boundary, which limits our understanding of their distribution during this period (Tintori et al., 2014; Ni et al., 2019). To date, fossil records of *Birgeria* are absent from the Neo-Tethys realm in the Late Triassic.

Herein, we report a suite of actinopterygian teeth with cutting edges that were discovered from middle Norian strata of the Tulong section, southern Tibet. The slightly curved teeth, consisting of an acrodin cap and a base with tiny vertical striae, are characteristic for those of *Birgeria*, thereby indicating the presence of large predatory actinopterygians within this portion of the Neo-Tethys during the Late Triassic.

## Geological setting

**Figure 1:**
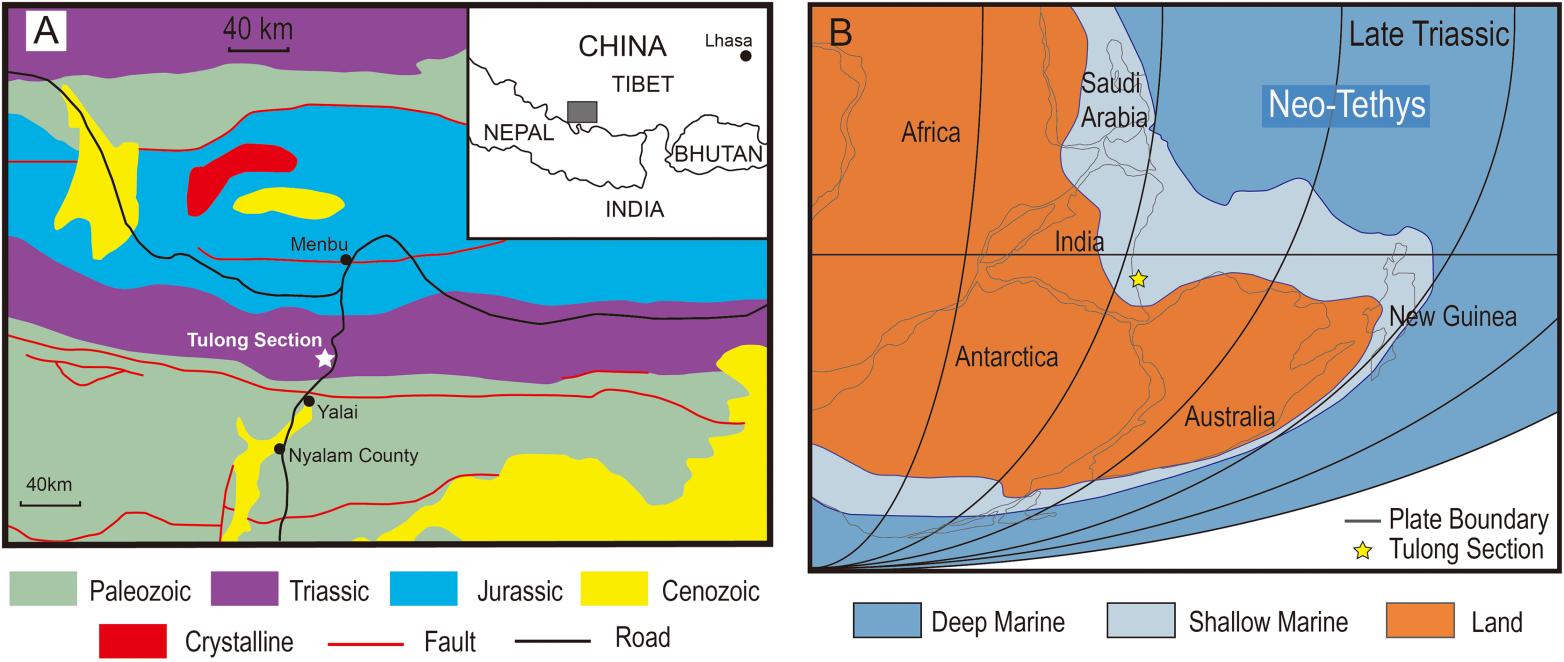
The geologic and paleogeographic map of the Tulong section. (A) Geologic map based on An et al. (2020) and the location of the Tulong section; (B) reconstructed paleogeographic position of the Tulong section, modified after Cai et al. (2016) with permission from Elsevier.

The Tulong section is located near Tulong Village, about 40 km northeast of Nyalam County in southern Tibet. Paleozoic and Mesozoic marine strata are widely distributed in this area (Fig. 1A). During the Triassic, the Tulong section was situated near the coast of central Gondwana on the passive margin of the Indian Plate facing the Neo-Tethys (Fig. 1B; *e.g*., Ogg & von Rad, 1994). The Upper Triassic of the Tulong section is mainly constituted by the Qulonggongba Formation and the Derirong Formation. The Qulonggongba Formation is dominated by clastic rocks intercalated with some carbonate rocks that were deposited in a shallow-marine environment (Zou et al., 2006; Cai et al., 2016). The overlying Derirong Formation is dominated by quartz sandstone (Meng et al., 2019). The enigmatic ichthyosaur, *Himalayasaurus tibetensis* (Dong, 1972), was found in the Qulonggongba Formation from this section, however the exact horizon was unclear.

**Figure 2:**
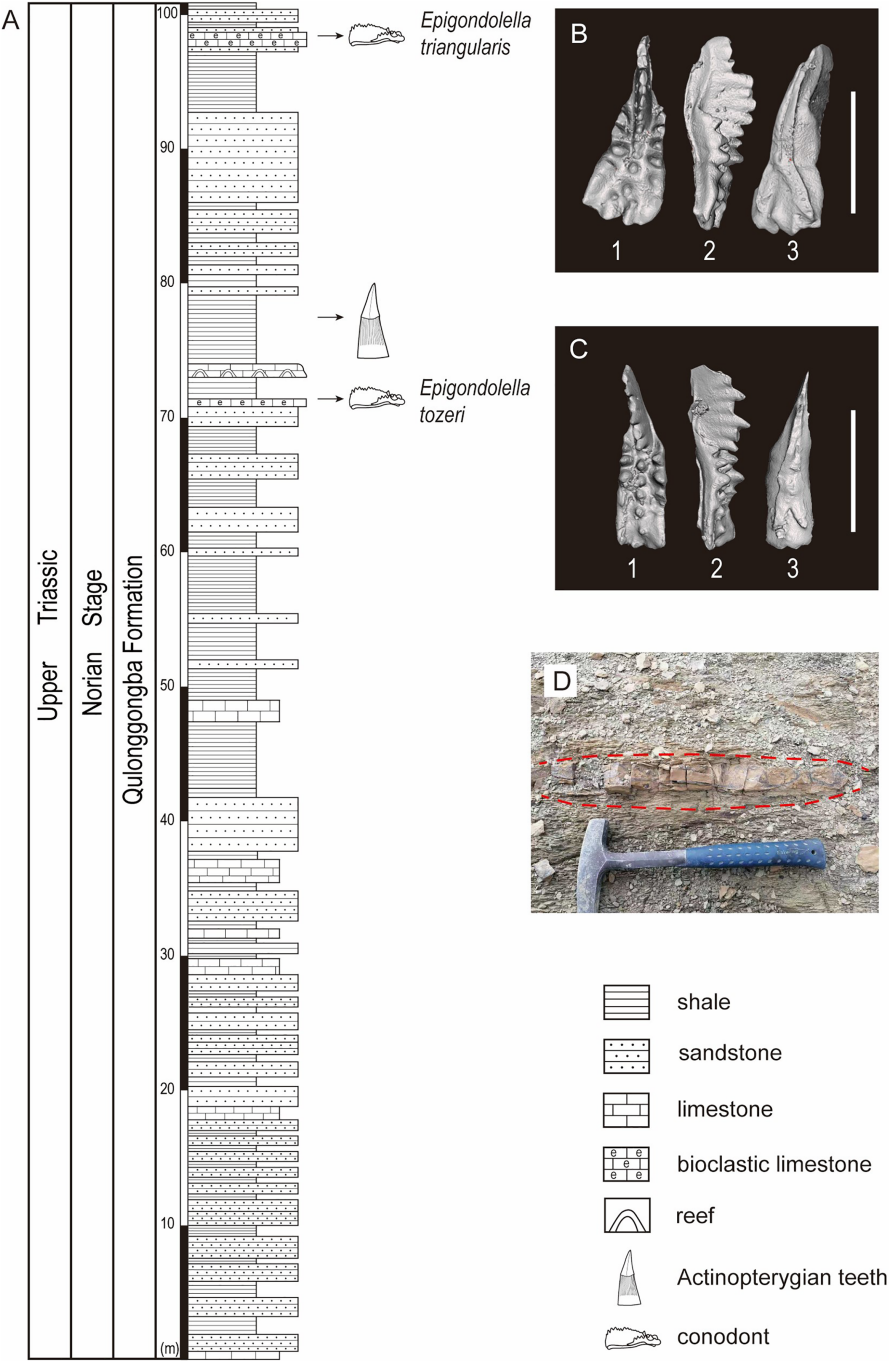
Stratigraphic profile of the Qulonggongba Formation at the Tulong section. (A) The stratigraphic column showing lithologies and fossil horizons in this study; (B) pictures of conodont *Epigondolella triangularis*; (C) pictures of conodont *Epigondolella tozeri*; (D) photo of the layer yielding the actinopterygian teeth fossils. In the conodont images, (1) upper view; (2) lateral view; (3) lower view; and the scale bar equals 0.5 mm.

The studied lower part of the Qulonggongba Formation is mainly dominated by grey shale and green and yellow sandstone, with some interbedded grey medium-bedded limestones (Fig. 2). Numerous fossils, including ammonoids, conodonts, bivalves and brachiopods were reported from the Qulonggongba Formation and indicate a Norian age (Zou et al., 2006).

In this study, we report a set of ray-finned fish teeth from a shale at the 75-m level in the measured Tulong section (Figs. 2A and 2D). Fortunately, we were able to separate the conodonts, *Epigondolella tozeri* (Orchard, 1991) and *Epigondolella triangularis* (Budurov, 1972), in co-occurrence with the fossilized teeth (Figs. 2A–2C). *E. tozeri* is known to occur in the Alaunian substage (middle Norian) (Karádi et al., 2021). *E. triangularis* has its first appearance during the latest Tuvalian (late Carnian) and can extend into early Alaunian, as documented both in the Tethys and in North America (Rigo et al., 2018). Therefore, our conodont materials suggest an assignment of a middle Norian age to the new tooth fossils from the studied section (Figs. 2B and 2C). A detailed biostratigraphy of the entire studied Tulong section will be the topic of a future article.

## Material and method

The assemblage of actinopterygian tooth fossils was discovered in the siliciclastic strata at the 75-m horizon (Fig. 2A). All of the teeth were prepared by carefully using a fine air chisel (brand: Hardy Winkler) and hand-held needles and then photographed. The statistics of the total length, the height of the apical cap, the basal diameter of the apical cap, and the maximal width are measured with a vernier caliper (precision 0.01 mm) for every element (Table 1). We follow the terminology for orientation and anatomical notation introduced by Cross et al. (2018).

**Table 1:** Measurements of teeth in this study with abbreviations.

(mm)	CUGW-TLV-1	CUGW-TLV-2	CUGW-TLV-3	CUGW-TLV-4	CUGW-TLV-5	CUGW-TLV-6	CUGW-TLV-7	CUGW-TLV-8	CUGW-TLV-9	CUGW-TLV-10	CUGW-TLV-11	CUGW-TLV-12
TL	7.00	17.09	18.96	16.32	6.80	8.44	—	—	10.99	—	—	12.05
HAC	3.11	6.08	7.82	—	—	5.19	7.66	7.03	7.85	8.76	6.63	—
BDC	1.63	3.68	4.28	4.37	3.44	3.00	—	—	4.02	—	3.40	3.84
MW	2.36	5.79	8.19	9.81	3.44	3.61	4.20	4.12	4.57	4.37	—	4.25

**Note:**

TL, the total length; HAC, the height of the apical cap; BDC, the basal diameter of the apical cap; MW, the maximal width.

The details of the conodonts and of the morphology of the actinopterygian teeth were revealed through Micro-CT examinations carried out in the Micro-CT Lab at the State Key Laboratory of Biogeology and Environmental Geology, China University of Geosciences (CUG), Wuhan, and a Bruker-MicroCT system-a SkyScan1172F (referred from Zhang et al., 2017). The X-ray source voltage and current were set at 49 kV/49 μA for tooth CUG-TLV-12 with a pixel resolution of 20.63 μm; 66 kV/151 μA for both two conodonts with a pixel resolution of 1.29 μm. The 3D images of conodonts and tooth CUG-TLV-12 were generated using CTvox. All specimens and their MicroCT data are housed in the School of Earth Sciences, CUG, (Wuhan). The 3D data of tooth CUG-TLV-12 and the two conodonts are deposited at MorphoSource (see Data Availability section).

**Figure 3:**
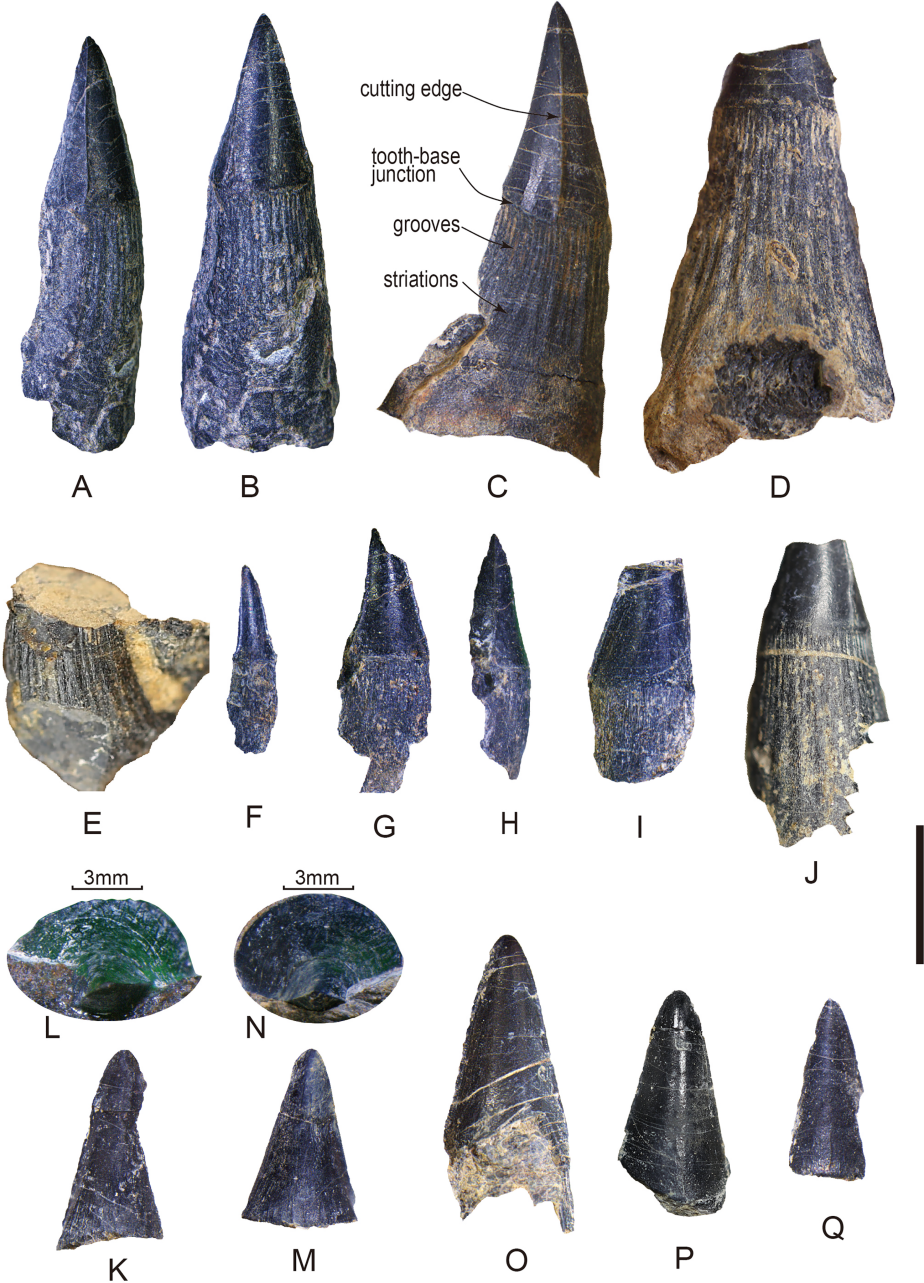
Tooth fossils from the Tulong section. (A) Tooth CUG-TLV-2 in lateral view; (B) CUG-TLV-2 in lingual view; (C) CUG-TLV-3; (D) CUG-TLV-4; (E) CUG-TLV-13; (F) CUG-TLV-1; (G) CUG-TLV-6 in labial view; (H) CUG-TLV-6 in lateral view; (I) CUG-TLV-5; (J) CUG-TLV-12; (K) CUG-TLV-7; (L) close-up of CUG-TLV-7 from apical view; (M) CUG-TLV-8; (N) close-up of CUG-TLV-8 from apical view; (O) CUGW-TLV-9; (P) CUG-TLV-10; (Q) CUG-TLV-11. Scale bar equals 5 mm.

Two histological thin sections were taken from the tooth CUG-TLV-12 in the vertical plane and tooth CUG-TLV-13 at the apical of the tooth base, respectively (Figs. 3E, 3J). The tooth CUG-TLV-13 was processed into a longitudinal section to examine the inner structure and the black fragment near the tooth base. The method of section preparing was referred from Ren et al. (2022), which the sample was embedded in Araldite-2020 one-component resin, cut with an STX-202A diamond wire automatic microtome. The sections were ground to a thickness of 100 μm in CUG-TLV-13 and 70 μm in CUG-TLV-13, with P400, P800, P1000 and P2000 abrasive article. The polished thin sections were observed and photographed under a ZEISS Primotech optical microscope (type Scope. A1) in normal transmitted light and polarized light, with 25x and 50x view, respectively.

## Description

Of the thirteen preserved teeth, four are nearly complete (CUG-TLV-1, 2, 3, and 6; Figs. 3A–3C, 3F–3H), four lack a complete apical cap of the tooth (CUG-TLV-4, 5, 12 and 13; Figs. 3D–3E, 3I–3J), and the other five only preserve the apical cap (CUG-TLV-7, 8, 9 10 and 11; Figs. 3K–3Q). In the four complete teeth, the total length is divided into a small-sized group (7 and 8.4 mm) and large-sized group (17.1 and 19.0 mm). The apical cap heights are 3.1, 5.19, 6.1 and 7.8 mm for the complete teeth (Table 1). Generally, the ratio of the height of the apical cap/the total height is 0.44, 0.35 and 0.41 in the complete teeth CUG-TLV-1, CUG-TLV-2 and CUG-TLV-3, respectively (Table 1).

We use the basal diameter of the apical cap (BDC) to assess the size of the incomplete teeth. In tooth CUG-TLV-4, the BDC reaches 4.4 mm, which is slightly larger than for CUG-TLV-3, thereby indicating that it is the largest size among these teeth (Fig. 3D). The diameter data for CUG-TLV-5 and CUG-TLV-12 are 3.4 and 3.8 mm, respectively, therefore the two teeth are slight smaller than CUG-TLV-3. Tooth CUG-TLV-13 had lost the apical region and the size is slightly larger than CUG-TLV-12 by visual observation. Despite five specimens preserving only the cap part, the height data enable comparing their approximate size. The arrangement from largest to smallest is CUG-TLV-9 (7.85 mm), CUG-TLV-10 (7.76 mm), CUG-TLV-7 (7.66 mm), CUG-TLV-8 (7.03 mm) and CUG-TLV- 11 (6.63 mm) (Table 1).

The discovered teeth are from the same horizon and are similar in general appearance. The complete teeth consist of elongated base with a compressed apical cap. The polished apical cap is slightly recurved lingually with a pointed apex in lateral view (Figs. 3A–3C). The cutting edges are continuous and smooth, beginning from the apical part of the cap and disappearing across the cap-base junction (Figs. 3A–3C). The cutting edges in the new teeth are less pronounced than those in the teeth of ichthyosaur *Himalayasaurus tibetensis*, which is found in the same section (Motani, Manabe & Dong, 1999). The cutting edges are present even in the smallest tooth CUG-TLV-1 (Fig. 3E). The specimens comprised only of the isolated cap are preserved in labial or lingual views with a rounded apex (Figs. 3L–3Q). In CUG-TLV-7 and CUG-TLV-8, the tip of the cap is flattened from the apical view, which is a typical characteristic in cutting teeth (Figs. 3L and 3N). The cap-base junction is distinctly straight.

The bases of the teeth are robust with numerous apicobasal grooves and striations. The lower margin of the apical cap is swollen slightly larger than the base in diameter. In the tooth base part, about twenty grooves at the lateral side are revealed in tooth CUG-TLV-3, terminating to the cap-base junction (Fig. 3C). The longitudinal grooves in tooth base are indented close to the cap-base junction, and gradually become shallow and wide striations with the tooth base growing downward. In some small teeth (CUG-TLV-1, CUG-TLV-5 and CUG-TLV-6), the longitudinal grooves of the tooth bases are shallow and ambiguous (Figs. 3E–3H). With the increasing size of the teeth, the grooves become clear and deep, such as in CUG-TLV-4 and CUG-TLV-12 (Fig. 3D and 3I).

**Figure 4:**
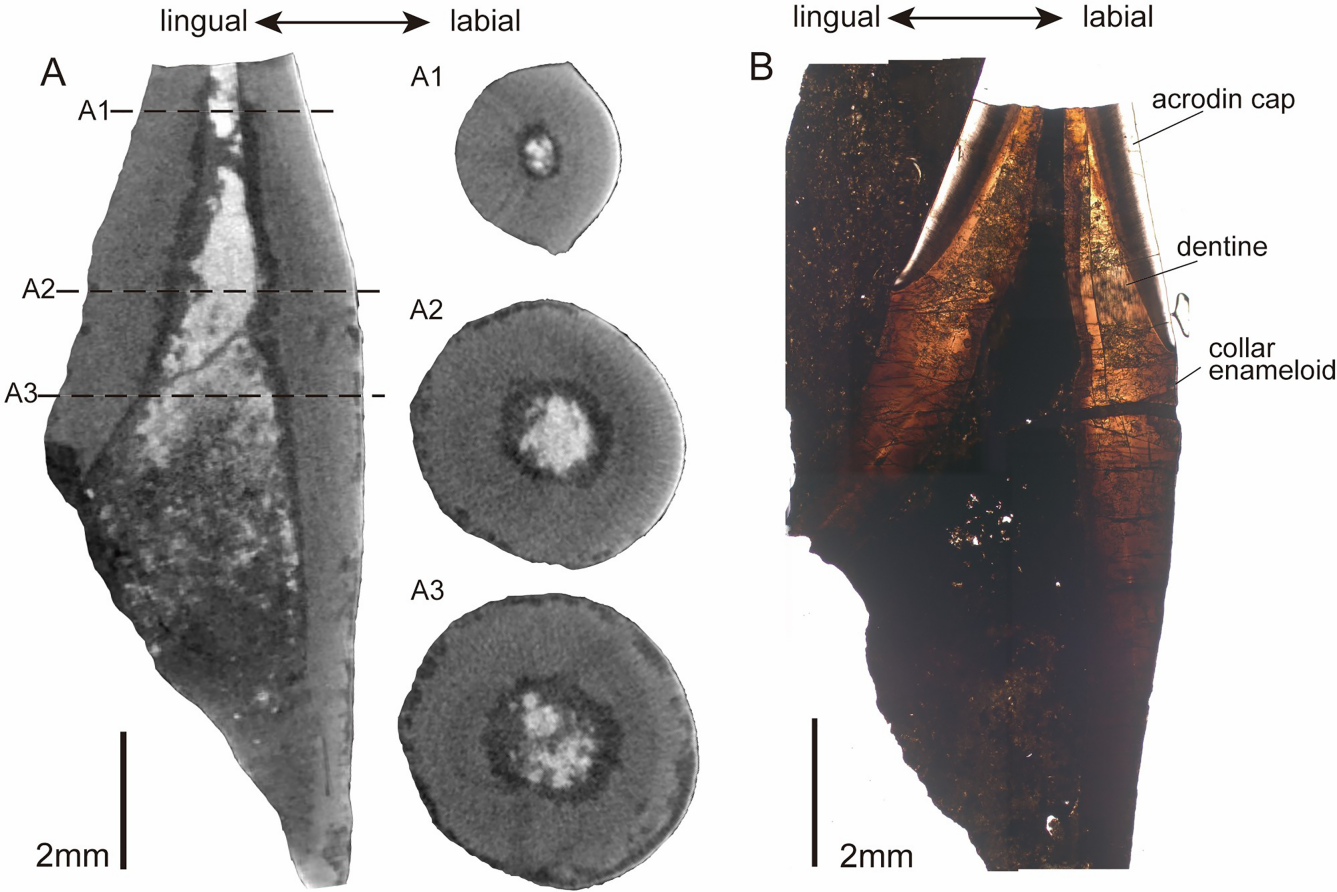
CT images and histological structure of the CUG-TLV-12. (A) The sagittal section along the labiolingual side, with the dotted lines indicating the location of the cross-sections (A1, A2, and A3); (A1) cross-section of the acrodin cap; (A2) cross-section near the cap-base junction; (A3) cross-section of the tooth base. (B) The histology of the vertical plane in normal transmitted light. Scale bar equals 2 mm.

In the horizontal cross-section of CUG-TLV-12, the outline of the apical cap is tear-shaped and divided into labial and lingual parts by paired cutting edges (Fig. 4A1). Distally from the crown, the outline of the cross-section transforms with a gradual rounding in the base (Fig. 4A2 and A3). In the vertical section, the pulpal cavity enlarges from the cap part to the base (Fig. 4A). The pulpal cavity extends a large part into the crown, measuring 3.85 mm in length and 0.72 mm in width. There are loose matters that fill the basal portion of the pulpal cavity of the CUG-TLV-12, which appear to represent the pulpal cementum of the replacement tooth. The vertical historical section shows two distinct layers in the apical cap: an inner tubular dentine and an outer enameloid layer (Fig. 4B). The enameloid layer has a translucent outer margin and massive collagen fibers near the inner margin, distinguished by the true “enamel” in amniotes (Sasagawa et al., 2009). The enameloid layer at the tip of the tooth is usually found in actinopterygians, and is called the “acrodin cap” (Sasagawa et al., 2008; Kawasaki et al., 2021). In the base region of the tooth, the collar enameloid layer is rather thin, which covers the surface of the dentine (Fig. 4B).

**Figure 5:**
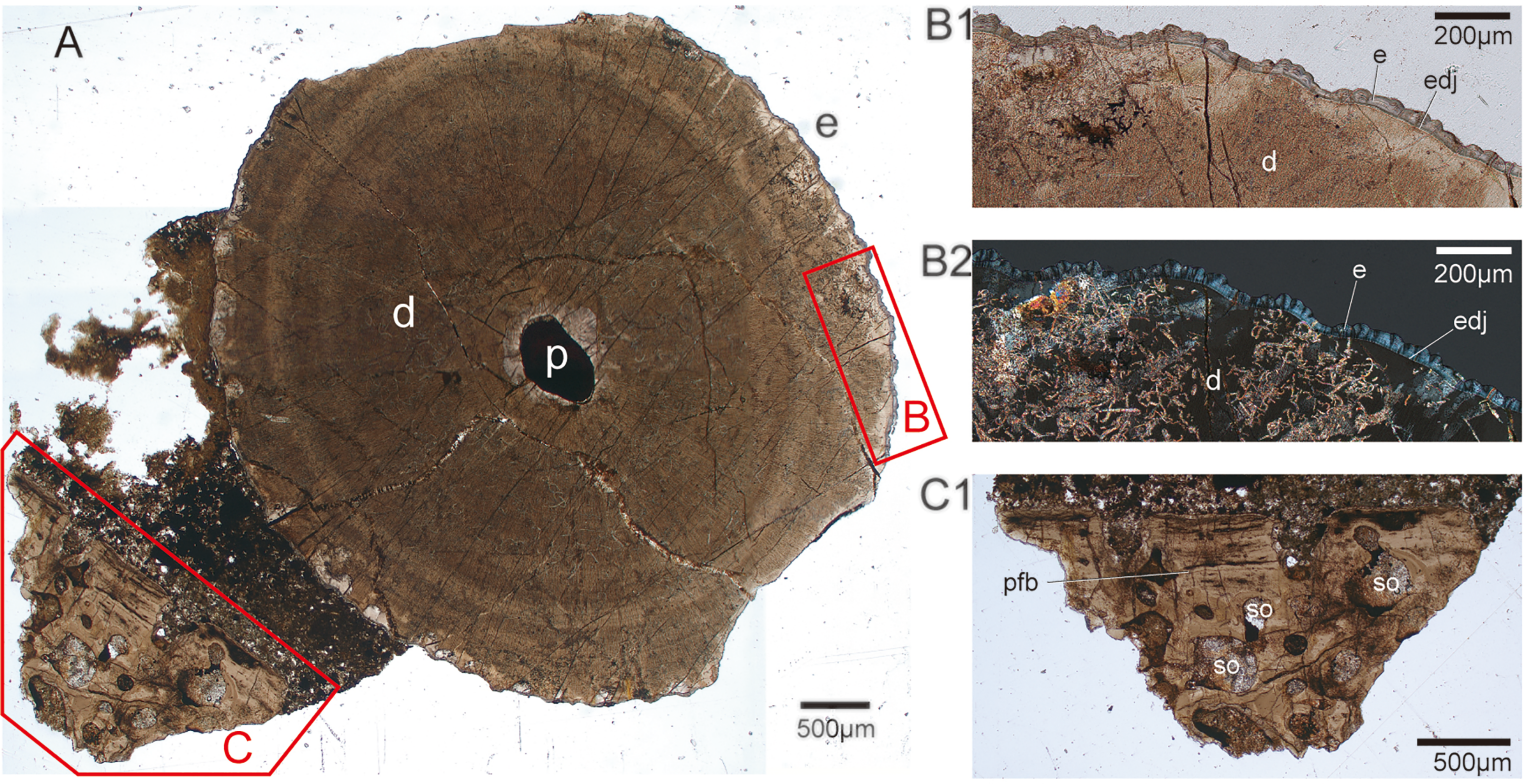
Transverse histological section taken from tooth CUG-TLV-13. (A) Transverse cross-section in normal transmitted light, showing the apical part of the tooth root with jaw fragment in CUG-TLV-13, and the enamel and the jaw area highlighted by the red-outlined boxes, (B) and (C), respectively; (B1) close up of the enamel area from the box B in normal transmitted light; (B2) close up of the enameloid area from the box B in polarized light; (C1) close up of the jaw area from the box C in normal transmitted light. Abbreviations: d, dentine; e, enameloid; edj, enameloid -dentine junction; p, pulp; pfb, parallel fibred bone; so, secondary osteon.

In the histological cross section of tooth CUG-TLV-13, the tooth base has a dentine and collar enameloid layer surrounding a dark-colored pulp cavity filled by non-transparent mineral (Fig. 5A). The dentine with radially arrayed dentinal tubules has a wavy margin and at least two light bands (Fig. 5A). Microstructural degradation in the dentine resembles microborings attributed to post-depositional bacterial or fungal activity (Fig. 5A), which commonly occur on fossil fish teeth (Underwood, Mitchell & Veltkamp, 1999; Weryński, Błażejowski & Kędzierski, 2023). The dentine recrystallization along the dentine-enameloid junction is probably caused by microborings (Fig. 5B1 and B2). The collar enameloid is mineralized and acellular, divided by a distinct dentine-enameloid junction along the dentine outer margin (Fig. 5B1 and B2). The collar enameloid thickens and produces crenulations upon the convex dentine (Fig. 5B1 and B2). The bone near the tooth could be a jaw fragment. The jaw bone towards the tooth is composed by parallel fibred tissue, forming a straight margin with invaginated foramina (Fig. 5C1). Secondary osteons are surrounded by collagen and are closely arranged (Fig. 5C1). While plicidentine is usually observed in some actinopterygian fishes, no evidence of its presence is found with either micro-CT or histology in new fossil teeth (Figs. 4A3, 5A; Maxwell, Caldwell & Lamoureux, 2011; Germain & Meunier, 2017).

## Discussion

### Taxonomic assignment of the new fossil teeth

The presence of the acrodin cap in the new teeth suggests an affinity with actinopterygian teeth, as evidenced by both their external and histological morphology. In their exterior, the new materials have an acrodin cap on the capital of the teeth and a slightly curved tooth base ornamented with subvertical fine striae, and these two parts have a distinct boundary, similar to actinopterygian teeth (Slater et al., 2016; Romano et al., 2017; Kowalski et al., 2019; Tayler et al., 2023). The histological structure of tooth CUG-TLV-12 indicates that the acrodin cap consists of a translucent matrix with massive collar fibers near the dentine margin (Sasagawa et al., 2009; Weryński, Błażejowski & Kędzierski, 2023). The acrodin cap is enameloid in actinopterygian teeth, which is distinct from the true “enamel” in tetrapod teeth (Ørvig, 1978; Sasagawa et al., 2008, 2009; Kawasaki et al., 2021).

Based on their morphology and stratigraphy, the new teeth could be assigned to actinopterygian fish of Norian age. The features of cone shape and cutting edges would exclude an affinity with some durophagous fish, such as *Sargodon* (Plieninger, 1847) and *Lepidotes* (Agassiz, 1832), which have blunted or molariform teeth (Tintori, 1998; Cross et al., 2018; de Lange et al., 2023). The new teeth resemble those of *Birgeria acuminata* (Agassiz, 1835–1844), *Saurichthys longidens* (Agassiz, 1835–1844) and *Gyrolepis albertii* (Agassiz, 1835–1844), which are commonly recognized within Norian and Rhaetian fish bonebeds, but the new teeth exhibit some differences with two latter taxa (Cross et al., 2018; Diependaal & Reumer, 2021; Tackett, Zierer & Clement, 2023; de Lange et al., 2023). The teeth in *Gyrolepis albertii* are elongated and slender, and have a small and straight acrodin cap that is about 10% of the total tooth length, which is contrary to our materials (Mears et al., 2016; de Lange et al., 2023). The teeth of *Birgeria* and *Saurichthys* are rather similar and were once considered to belong to a single *Severnichthys* taxon (Storrs, 1994). However, Diependaal & Reumer (2021) argued *Severnichthys* as a *nomen dubium* with a type material probably representing in fact *Birgeria*. We follow this conclusion herein. The main features suggested to distinguish the two morphotypes are: (a) the acrodin cap in *Birgeria* makes up more of the overall length than that in *Saurichthys*; (b) the teeth in *Birgeria* have a prominent circumferential ridge around the base of the acrodin cap, but the same ridge is ambiguous in *Saurichthys*; (c) the ridges and grooves in tooth base are strongly indented in *Saurichthys*, otherwise the tooth base is often ornamented by numerous fine striations in *Birgeria*; (d) the pulp cavity extends deeply above the base of the acrodin cap in *Birgeria* than in *Saurichthys*; and (e) the plicidentine in the tooth base is present in *Saurichthys* but absent in *Birgeria* (Błażejowski et al., 2013; Romano et al., 2017; Diependaal & Reumer, 2021; Tackett, Zierer & Clement, 2023; de Lange et al., 2023; Evans et al., 2024). According to the comparison above, the new teeth are more likely to belong to *Birgeria* rather than to *Saurichthys*.

### Paleobiogeographic implications of the new teeth

*Birgeria* were pelagic fish and spanned almost the entire Triassic period with a global distribution, but their fossil record is rare in the southern hemisphere (Romano et al., 2017; Ni et al., 2019). During the Early Triassic, four species of *Birgeria* emerged immediately after the End-Permian Mass Extinction, of which three of them were known only from East Greenland (*Birgeria groenlandica*; Stensiö, 1932), Spitzbergen (*Birgeria aldingeri*; Schwarz, 1970) and Nevada (*Birgeria americana*; Romano et al., 2017). *Birgeria nielseni* (Lehman, 1948), the only valid taxon from the southern hemisphere, was named based on a detailed description of a specimen from the Lower Triassic of Madagascar (Lehman, 1952; Ni et al., 2019). Additionally, fossil fragments were found in Canada and Russia (Berg, Kazantseva & Obruchev, 1967; Schaeffer & Mangus, 1976; Ni et al., 2019). Besides that occurrence, some fragments of *Birgeria* were reported from the Vitiacua Formation of Bolivia, with a questionable age from the Late Permian to the Early Triassic (Beltan et al., 1987; Cione et al., 2010; Romano et al., 2017).

Entering the Middle Triassic, *Birgeria* contained four species, identified in the United States (*Birgeria velox*; Jordan, 1907), China (*Birgeria liui*; Jin, 2001), Germany (*Birgeria mougeoti*; Agassiz, 1834) and Switzerland (*Birgeria stensioei*; Aldinger, 1931), respectively (Ni et al., 2019). Many presumable teeth and a fragmentary caudal fin of indetermined *Birgeria* sp. have been found in the northern hemisphere, such as Austria (Krainer, Lucas & Strasser, 2011), Belgium (Duffin & Delsate, 1993), Poland (Chrząstek, 2008) and China (Jiang et al., 2016). But teeth of *Birgeria* sp. were discovered in two southern paleo-hemisphere locations, India and Saudi Arabia, thereby indicating the existence of this fish genera within the Gondwana region during this epoch (Chhabra & Mishra, 2002; Vickers-Rich et al., 1999).

During the Late Triassic, the known diversity of *Birgeria* had declined to only two species, with *Birgeria guizhouensis* (Liu et al., 2006) reported in the Lower Carnian from China and *Birgeria acuminata* being the only species recognized in the Norian to Rhaetian strata from Europe (Tintori & Lombardo, 2018). An abundance of teeth of *Birgeria* were discovered along with several fish fauna from the famous “Rhaetian Bone Beds” of England and Europe (Slater et al., 2016; Mears et al., 2016; Cross et al., 2018; Williams et al., 2022; Szabó, Botfalvai & Ősi, 2019; Diependaal & Reumer, 2021; de Lange et al., 2023; Evans et al., 2024). *Birgeria* has been recorded in Norian marine deposits of Canada (Orchard et al., 2001), the United States (Tackett, Zierer & Clement, 2023) and possibly Bolivia (Beltan et al., 1987). In a sense, the distribution *Birgeria* during Late Triassic seems mainly concentrated to the western Paleo-Tethys and Euramerican Realm and retreated from the Neo-Tethys according to the present literature. Therefore, the discovery of the teeth of *Birgeria* in the Tulong section of southern Tibet, which was formerly the northern margin of the Indian Plate, clearly indicates that this fish was still active in the Neo-Tethyan region at least into the Norian period, and its paleobiogeographic distribution might be more complex than previously thought (Tintori et al., 2014; Tintori & Lombardo, 2018).

**Figure 6:**
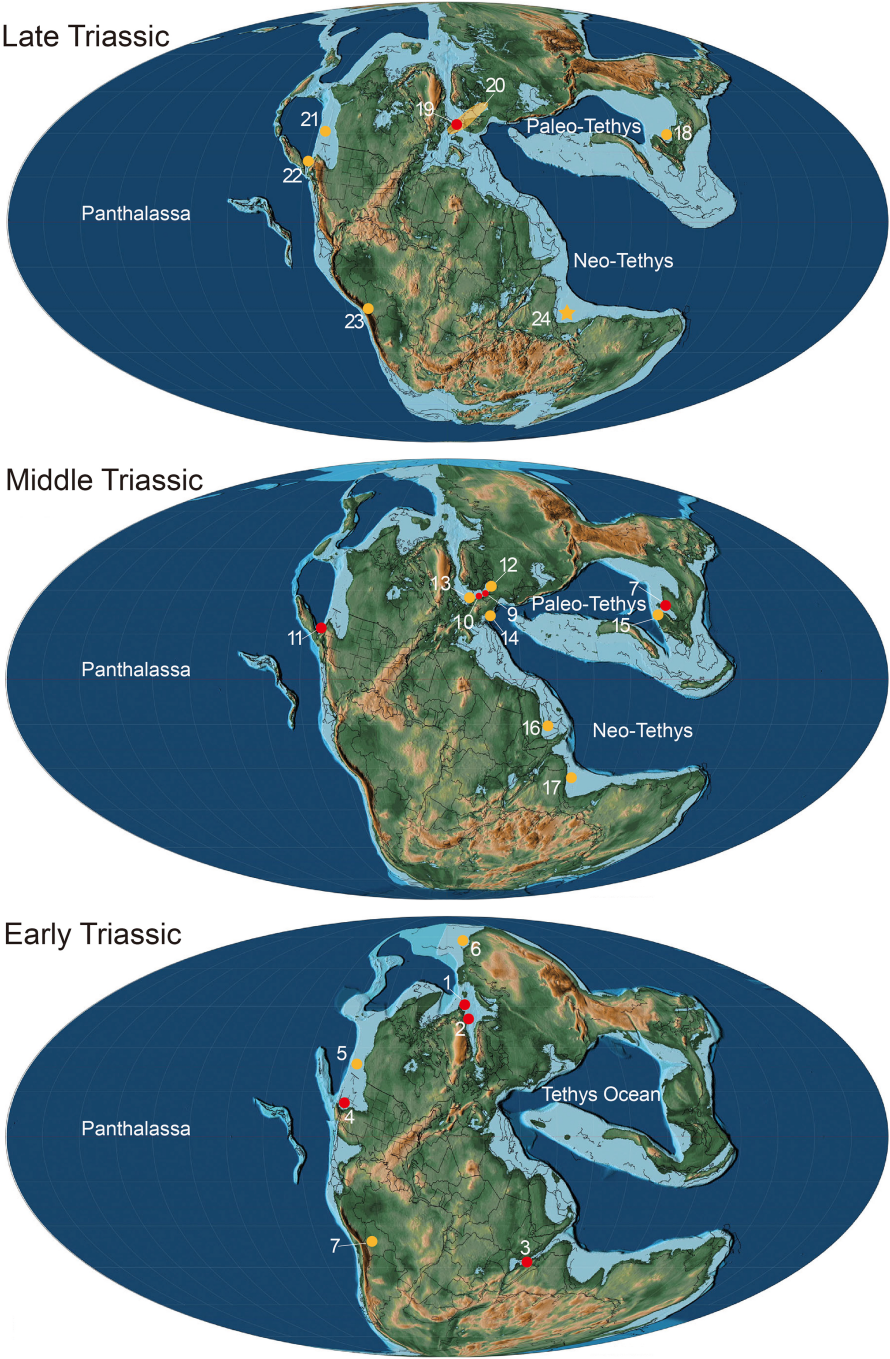
Paleogeographical distribution of *Birgeria* in the Triassic. Paleogeographic map adopted from Scotese (2014) © PALEOMAP Project (http://www.scotese.com/), records of *Birgeria* mostly adopted from Ni et al. (2019) with modification. Red dots mean the fossil records with valid species, and yellow dots mean that with bony fragments. Early Triassic period: 1. *Birgeria aldingeri*, Spitzbergen; 2. *B. groenlandica*, Greenland; 3. *B. nielseni*, Madagascar; 4. *B. americana*, Nevada 5. fragments, British Columbia (Schaeffer & Mangus, 1976); 6. fragments, Siberia (Berg, Kazantseva & Obruchev, 1967); 7. fragments, Bolivia (Beltan et al., 1987). Middle Triassic period: 8. *B. liui*, South China; 9. *B. Stensiöi*, Switzerland; 10. *B. mougeoti*, Germany; 11. *B. velox*, California; 12. fragments, Poland (Chrząstek, 2008); 13. fragments, Belgium (Duffin & Delsate, 1993); 14. fragments, Austria (Krainer, Lucas & Strasser, 2011); 15. fragments, South China (Jiang et al., 2016); 16. fragments, Saudi Arabia (Vickers-Rich et al., 1999); 17, fragments, India (Chhabra & Mishra, 2002). Late Triassic: 18. *B. guizhouensis*, South China; 19. *B. acuminata*, Europe; 20. fragments, “Rhaetian Bone Beds” and other European fossil occurrences (Tintori & Lombardo, 2018); 21. fragments, Canada (Orchard et al., 2001); 22. fragments, Nevada (Tackett, Zierer & Clement, 2023); 23. fragments, Bolivia (Beltan et al., 1987); 24. fragments, Tulong section, Tibet, in this study.

Our findings indicates that *Birgeria* maintained its distributions in the southern hemisphere during the whole Triassic, both the coastline of the Panthalassa and Neo-Tethys realm (Fig. 6). The distribution of *Birgeria* fossil fragments is more widespread than that of valid species. The cosmopolitan *Birgeria* kept low diversity throughout the Triassic, contrasted with its relative, *Saurichthys*, which shows both high distribution and diversity (Romano et al., 2013; Tintori et al., 2014; Ni et al., 2019). Although it is a fact that *Birgeria* is rarer than *Saurichthys*, preservation or sampling bias may further limit the recognitions of *Birgeria*. Tintori et al. (2014) suggested a gap in the fossil record of marine fish between the early Carnian and middle/late Norian boundary, as well as the absence of Late Triassic fish fauna from the Neo-Tethys region. The existence of *Birgeria* from the Neo-Tethys with a definite middle-Norian age fills the gap and suggests that the diversity drop of fish diversity in the Late Triassic is probably affected by preservation and hence collection bias (Romano et al., 2013; Tintori & Lombardo, 2018; Rigo et al., 2020).

### The paleo-ecological impact of *Birgeria*

*Birgeria* were large fast-swimming predatory actinopterygians that possessed a strong and blunt rostrum with cutting-edged teeth, and are considered to have occupied higher trophic levels throughout the Triassic period (Scheyer et al., 2014; Romano et al., 2017; Tintori & Lombardo, 2018). The presence of anterior and posterior cutting edges on the teeth in all three rows indicates they were a carnivorous predator (Romano et al., 2017); these cutting edges are also preserved in our new materials. While it is difficult to estimate the length of complete individuals by only the tooth length, a limited comparison could be made with fragmentary remains. The new teeth are much larger than the small-sized *Birgeria* teeth from the Norian and Rhaetian fauna (Cross et al., 2018; Diependaal & Reumer, 2021; Tackett, Zierer & Clement, 2023). The largest tooth from the new materials is comparable in length to those of the large-sized *Birgeria americana*, thereby suggesting that the owner(s) of the new teeth may have had a total length reaching up to 1 m (Romano et al., 2017). Their relatively large body size and sharp teeth help make *Birgeria* become efficient predators, which usually fed on durophagous fishes or even small-sized marine reptiles (Cross et al., 2018; Tackett, Zierer & Clement, 2023; Quinn & Matheau-Raven, 2024). However, in the paleoecology of the Tulong section, *Birgeria* could be overtaken by the co-occurring apex ichthyosaur predator, *Himalayasaurus tibetensis*, which has dagger-like cutting teeth and a very large body size that is over 10 m (Motani, Manabe & Dong, 1999).

Our findings show that predatory actinopterygian *Birgeria* existed during the middle Norian in this region of the Neo-Tethys in the southern paleo-hemisphere. Therefore, predators filling different niches were foraging in the southern Tethys Ocean during this time. This implies that the marine ecosystems provided sufficient nutrition and energy to supply these apex predators prior to the late Norian wave of extinctions (Rigo et al., 2020). The biotic fossil assemblages at the Tulong section mainly consist of invertebrates, such as ammonites and bivalves, of conodonts and of chondrichthyes cartilaginous fish, such as Hybodontidae (Dong, 1972; Zou et al., 2006), but are lacking primary consumers, such as durophagous fishes and other tetrapods. Therefore, future research in the Tulong and other sections should focus on the paleobiology and paleo-ecosystems of this region.

## Conclusion

Thirteen isolated teeth fossils were discovered which reveal an affinity with actinopterygians, and the conodonts in this same horizon indicate a middle Norian age. The remarkable morphology of the new teeth, such as the polished acrodin cap, the straight cap-base ridge and the conic shape with two cutting edges, is similar to those of the large actinopterygian *Birgeria*. The owner(s) of the new teeth, in co-occurrence with the ichthyosaur *Himalayasaurus tibetensis*, had the role of a carnivorous predator. This is their first report from the Neo-Tethyan realm of the southern paleohemisphere. The biotic assemblages in the Tulong section mainly consist of invertebrates and predators, but lack a fossil record of the primary vertebrate consumers. Therefore, further investigation should be directed toward reconstructing the paleo-ecological network of this Neo-Tethys setting.

## Additional Information and Declarations

### Data Availability

The following information was supplied regarding data availability: The 3D data of the specimen CUG-TLV-12 and two conodonts are available at MorphoSource: - *Epigondolella tozeri*, 000491867, https://doi.org/10.17602/M2/M491867; - *Epigondolella triangularis*, 000491872, https://doi.org/10.17602/M2/M491872; -The specimen CUG-TLV-12, 000491776, https://doi.org/10.17602/M2/M491776.
